# The *Arabidopsis thaliana MHX* gene includes an intronic element that boosts translation when localized in a 5′ UTR intron

**DOI:** 10.1093/jxb/ert235

**Published:** 2013-09-04

**Authors:** Tsofit Akua, Orit Shaul

**Affiliations:** The Mina and Everard Goodman Faculty of Life Sciences, Bar-Ilan University, Ramat-Gan, Israel

**Keywords:** IME, intron-mediated enhancement, leader intron, translational regulation, 5′ untranslated region, 5′ UTR.

## Abstract

The mechanisms that underlie the ability of some introns to increase gene expression, a phenomenon called intron-mediated enhancement (IME), are not fully understood. It is also not known why introns localized in the 5′-untranslated region (5′ UTR) are considerably longer than downstream eukaryotic introns. It was hypothesized that this extra length results from the presence of some functional intronic elements. However, deletion analyses studies carried out thus far were unable to identify specific intronic regions necessary for IME. Using deletion analysis and a gain-of-function approach, an internal element that considerably increases translational efficiency, without affecting splicing, was identified in the 5′ UTR intron of the *Arabidopsis thaliana MHX* gene. Moreover, the ability of this element to enhance translation was diminished by a minor downstream shift in the position of introns containing it from the 5′ UTR into the coding sequence. These data suggest that some of the extra length of 5′ UTR introns results from the presence of elements that enhance translation, and, moreover, from the ability of 5′ UTR introns to provide preferable platforms for such elements over downstream introns. The impact of the identified intronic element on translational efficiency was augmented upon removal of neighbouring intronic elements. Interference between different intronic elements had not been reported thus far. This interference may support the bioinformatics-based idea that some of the extra sequence of 5′ UTR introns is also necessary for separating different functional intronic elements.

## Introduction

The ability of introns to boost gene expression was identified in many organisms, including plants. In most cases, the presence of introns increases the steady-state levels of mature mRNA in the cell (Callis *et al.*, 1987; Dean *et al.*, 1989; Rethmeier *et al.*, 1997; Nott *et al.*, 2003; Rose, 2004, 2008; Morello *et al.*, 2011). In both plants and mammals, introns do not enhance expression by increasing mRNA stability (Rethmeier *et al.*, 1997; Nott *et al.*, 2003). In certain cases, introns were shown to act as transcriptional enhancers or to include internal promoters (e.g. Furger *et al.*, 2002; Morello *et al.*, 2002; Samadder *et al.*, 2008). However, in many other cases, introns were shown to enhance expression without altering the rate of transcription initiation (e.g. Dean *et al.*, 1989; Rose and Last, 1997). The latter phenomenon was termed ‘intron-mediated enhancement’ (IME) (reviewed by Rose, 2008).

The ability of some introns to enhance gene expression without increasing the rate of transcription initiation is not fully understood. Intron splicing *per se* is not sufficient for enhancing expression, as evident from the fact that different introns have different abilities to boost expression, and some introns cannot stimulate expression at all. Yet, deletion analysis studies were unable to identify specific intronic regions necessary for IME (reviewed by Rose, 2008). For example, the ability to boost expression of the entire *AtUBQ10* intron was roughly the sum of the stimulation mediated by each of its parts (Rose *et al.*, 2008). Based on the observation that most enhancing introns are first introns, a bioinformatic approach was used to identify short sequence elements that distinguish promoter-proximal introns from distal *Arabidopsis* introns (Rose *et al.*, 2008). This resulted in assignment to introns of an ‘IMEter score’, which reflects the abundance of the indicated short sequences in individual introns (Rose *et al.*, 2008; Parra *et al.*, 2011). There was a good correlation between the IMEter scores of different introns and their ability to enhance mRNA accumulation in *Arabidopsis* (Rose *et al.*, 2008; Parra *et al.*, 2011). The short IMEter sequences were found to be redundant and dispersed throughout enhancing introns. This explained the inability to identify specific intronic regions necessary for IME by deletion analysis (reviewed by Rose, 2008). The mechanism(s) by which the IMEter sequences enhance mRNA accumulation is currently unknown. It was supposed that the IMEter sequences render the transcription machinery more processive, increasing the likelihood that full-length mRNAs will accumulate (Rose *et al.*, 2008; Parra *et al.*, 2011). There are also indications that IME may act at the level of DNA (Rose *et al.*, 2011). Besides the short IMEter sequences, a 35bp U-rich motif of the maize *Sh1* first intron was shown to increase expression without altering the efficiency of splicing (Clancy and Hannah, 2002). This motif could be replaced by another U-rich motif of the same intron, indicating that the important feature of this motif was U-richness rather than the specific sequence.

Introns also enhance mRNA accumulation through their impact on mRNA export from the nucleus to the cytosol. Upon splicing, the spliceosome deposits multiple proteins, known as the exon junction complex (EJC), 20–24 nucleotides upstream of exon–exon junctions (Le Hir *et al.*, 2000). It was shown that the EJC promotes efficient export from the nucleus of spliced mRNA (Le Hir *et al.*, 2001; Dimaano and Ullman, 2004; Valencia *et al.*, 2008). Overexpression of *Arabidopsis* homologues of some EJC components increased the expression of intron-containing reporter constructs, suggesting that the EJC also participates in IME in plants (Mufarrege *et al.*, 2011).

Besides increasing mRNA content, the presence of introns was also shown to increase the efficiency of mRNA translation in plants, mammals, and *Xenopus* (Mascarenhas *et al.*, 1990; Matsumoto *et al.*, 1998; Bourdon *et al.*, 2001; Nott *et al.*, 2003, 2004; Rose, 2004; Gudikote *et al.*, 2005; Curi *et al.*, 2005; Samadder *et al.*, 2008). The enhancing effect of introns on translation is also mediated by the EJC (Wiegand *et al.*, 2003; Nott *et al.*, 2004; Lee *et al.*, 2009; reviewed by Le Hir *et al.*, 2003; Moore and Proudfoot, 2009). Only two studies, carried out on human HeLa cells, gave some clues about how the EJC mediates the increased translational efficiency. A protein named PYM was shown to bridge the EJC and the 48S pre-initiation ribosomal complex (Diem *et al.*, 2007). S6K1, a kinase of an 40S ribosomal subunit protein, increases translation by binding the EJC-associated protein SKAR (Max *et al.*, 2008).

Bioinformatic studies identified that in many organisms first introns are longer than downstream introns (Bradnam and Korf, 2008). Moreover, 5′-untranslated region (5′ UTR) introns are in general considerably longer than first introns of genes that lack 5′ UTR introns (Bradnam and Korf, 2008). The reasons for this extra length are not fully understood. Calculations indicated that the IMEter sequences could not account for all of the additional length of first introns (Bradnam and Korf, 2008). It was therefore suggested that first introns may contain other functional elements (Bradnam and Korf, 2008). It was also suggested that some of the additional sequence might be necessary to provide spacing between different functional elements in order to avoid interference between proteins or RNAs that bind them (Bradnam and Korf, 2008). Bioinformatics also showed that *Arabidopsis* 5′ UTR introns are located closer to the AUG codon than to the 5′ end of the transcript (Chung *et al.*, 2006). Based on this observation, it was hypothesized that there may be an optimal distance between 5′ UTR introns and AUG codons, and that these introns may play a role in translation (Chung *et al.*, 2006). Yet, intronic elements that enhance translation have not been identified thus far, nor was there any experimental evidence for the significance of the position of such putative elements relative to the initiation codon.

The 416 nucleotide long 5′ UTR intron [or leader intron (LI)] of the *Arabidopsis AtMHX* gene (Shaul *et al.*, 1999; Berezin *et al.*, 2008a, *b*) was previously shown to substantially increase gene expression, without acting as a transcriptional enhancer (David-Assael *et al.*, 2006; Akua *et al.*, 2010). In the present work, analysis of this 5′ UTR intron resulted in the first identification of an internal intronic element that has a special ability to enhance translational efficiency. Moreover, this work demonstrates that the ability of this element to enhance translation is dependent on localization of introns containing it in the 5′ UTR, and is diminished by a minor downstream shift in the position of these introns into the coding sequence. This suggests that some of the extra length of 5′ UTR introns results from the presence of elements that enhance translation, and, moreover, from the ability of 5′ UTR introns to provide preferable platforms for such elements over downstream introns. This work also shows that the impact of the identified intronic element on translation is repressed by its neighbouring intronic elements. Interference between different intronic elements had not been reported thus far. This interference supports the bioinformatics-based idea that some of the extra sequence of 5′ UTR introns may also be necessary for separating different functional intronic elements.

## Materials and methods

### Plant transformation and growth conditions

*Arabidopsis thaliana* (L.) (ecotype Col-0) plants were transformed using *Agrobacterium* by the floral dip technique (Clough and Bent, 1998). Following selection on kanamycin, ~45 independently transformed T_1_ plants were obtained for each construct. The T_1_ plants were grown in the greenhouse and their T_2_ seeds were collected. For expression analysis, T_2_ seedlings germinated from mixtures including equal amounts of seeds from each of the 45 independent T_1_ plants of each construct were grown on MS plates containing kanamycin. The plants were grown for 2 weeks in a climate-controlled growth room in a photoperiod of 16h light and 8h dark.

### Generation of constructs

The generation of the WT and –I (–intron) constructs of the DEL series has been described (Akua *et al.*, 2010). The other DEL series constructs were created by amplification of construct WT in two reactions. In the first reaction, the forward primer was 5′-GATAATATCTAAACTCGAGGAGATGATAA-3′ and the reverse primer corresponded to the desired internal sequence of the LI. In the second reaction, the forward primer corresponded to the desired internal sequence of the LI and the reverse primer was 5′-TTAGGCCATGGTAACTTATTCAAA-3′. The product of the first reaction was digested with *Xho*I. The product of the second reaction was digested with *Nco*I, and treated with T_4_ kinase to allow ligation of the two PCR products. The two products were simultaneously cloned into the *Xho*I–*Nco*I sites of the WT construct.

To create the UTR series constructs, a *Bgl*II site was introduced into the WT construct at a downstream region of the *AtMHX* promoter, and a *Bgl*II site was eliminated from the coding sequence of *GUS* (β-glucuronidase, derived from the *Escherichia coli udiA* gene), without altering the deduced amino acid sequence. A DNA fragment carrying the downstream region of the *AtMHX* promoter, the 5′ UTR of this gene, and an intron from the *Ricinus communis (L.) CAT1* gene (GenBank accession no. D21161.1) replacing the LI in the 5′ UTR (at its precise location) was synthesized by GenScript (Piscataway, NJ, USA). The modifications introduced into this sequence (introduction of restriction enzyme sites, elimination of potential cryptic splice sites, and elimination of AUG triplets) are detailed in Supplementary Fig. S1 available at *JXB* online. In particular, a potential cryptic splice site located seven nucleotides before the 3′ end of the *CAT1* intron was eliminated here (Supplementary Fig. S1B at *JXB *online). It was shown later on that elimination of this potential cryptic splice site resulted in full and accurate splicing of the *CAT1* intron (Ma *et al.*, 2011). Prediction of potential cryptic splice sites was carried out using the NetPlantGene Server (http://www.cbs.dtu.dk/services/NetPGene/). The synthesized DNA fragment was used to replace the 5′ UTR and LI between the *Bgl*II site of the promoter and the *Mva*1269I site of *GUS*. Different elements of the modified LI of *AtMHX* (Supplementary Fig. S2 at *JXB *online), which was also synthesized by GenScript, were amplified by PCR and cloned into the middle of the *Hpa*I site of the *CAT1* intron, thereby creating the different UTR series constructs. To create the –I construct of the UTR and CDS series, the 5′ UTR of *AtMHX* with the necessary flanking sequences (the downstream region of the *AtMHX* promoter and the coding sequence of *GUS* up to the *Mva*1269I site) was cloned between the *Bgl*II site of the promoter and the *Mva*1269I site of *GUS*.

To create the CDS series constructs, a DNA fragment carrying the downstream region of the *AtMHX* promoter, the 5′ UTR of *AtMHX*, and the coding sequence of *GUS* up to the *Mva*1269I site was synthesized by GenScript. The *GUS* coding sequence included the *CAT1* intron 12bp downstream of the AUG codon (Supplementary Fig. S3 at *JXB* online). This DNA fragment was cloned between the *Bgl*II site of the promoter and the *Mva*1269I site of *GUS*. The different elements of the LI of *AtMHX* were digested from the corresponding UTR series constructs by *Nhe*I and *Afl*II, and introduced into the same sites of the *CAT1* intron, thereby creating the different CDS series constructs. All chimeric genes were verified by sequencing, cloned into the binary vector pGA492 (An, 1986), immobilized into *Agrobacterium* EHA105 (Hood *et al.*, 1993), and used for plant transformation.

### RNA extraction and northern blot analysis

Total RNA was extracted with TRI-Reagent (Sigma) according to the manufacturer’s instructions. RNA samples were denatured with glyoxal (Sigma) and fractionated on 1% agarose gels as described (Sambrook and Russell, 2001). Gel preparation and fractionation were carried out with 10mM NaPi buffer, pH 7.0. The gels were blotted onto a Zeta-Probe GT membrane (Bio-Rad) with 25mM NaPi buffer, pH 7.0. RNA was fixed by UV. The membranes were stained by 0.02% methylene blue in 0.3M sodium acetate (pH 5.5) to visualize the rRNA, and then rinsed in H_2_O. Hybridization was carried out using the DIG-labelling system (Roche Diagnostics GmbH) according to the manufacturer’s instructions. Quantification of band densities on gels was performed with the ImageJ program (NIH).

### Preparation of cDNA and reverse transcription–polymerase chain reaction (RT–PCR)

RNA was treated with DNase I, followed by DNase I removal, using Ambion’s AM1906 kit according to the manufacturer’s instructions. A preliminary PCR with the same primers that were subsequently used for RT–PCR was conducted to verify that no DNA remained. Preparation of cDNA was carried out using an oligo(dT) primer and M-MuLV reverse transcriptase (RevertAid™, Fermentas) according to the manufacturer’s instructions. A 1.5 μl aliquot of each cDNA was utilized as template for RT–PCR analysis with the following primers: forward primer 5′-GCAGGATCCACGCTTGACCGATTC-3′ (corresponding to the first exon of the 5′ UTR) and reverse primer 5′-TTCGCGATCCAGACTG-3′ (corresponding to ~100bp down stream of the initiation codon of *GUS*). PCR conditions were as follow: an initial cycle of 94 °C for 3min, followed by 30 cycles of 94 °C for 30 s, 53 °C for 30 s, and 72 °C for 2min, and a final cycle of 72 °C for 10min.

### Quantitative GUS analysis

Quantitative measurement of GUS activity was carried out using the fluorometric assay described by Breyne *et al.* (1993). This kinetic assay was shown to be more accurate than end-point measurements (Breyne *et al.*, 1993). Plant material was ground in liquid nitrogen and extracted in a buffer containing 50mM NaPO_4_, pH 7.2, 1mM Na_2_EDTA, 10mM β-mercaptoethanol, and 10% (v/v) Triton X-100. Following centrifugation (5min, 14 000 *g*, 4 °C), the supernatant was collected and the concentration of proteins was determined using the Bradford reagent (Sigma). Samples including equal amounts of protein were suspended in 250 μl of extraction buffer including 1mM (final concentration) of the fluorescent GUS substrate 4-methylumbelliferyl-β-d-glucuronide (MUG) (Duchefa Biochemie BV). GUS activity was assayed on a 96-well fluorescent plate-reader (Fluoroscan II, Lab Systems) with the excitation wavelength set at 350nm and the emission wavelength at 460nm. GUS activity (milli units mg protein^–1^) was calculated from the slope of the line generated from measurements taken at 3min intervals during 2h, with respect to the slope of commercial pure GUS enzyme (Roche Diagnostics GmbH).

## Results

### Identification of LI elements that affect expression independently of their impact on splicing

**Fig. 1.**
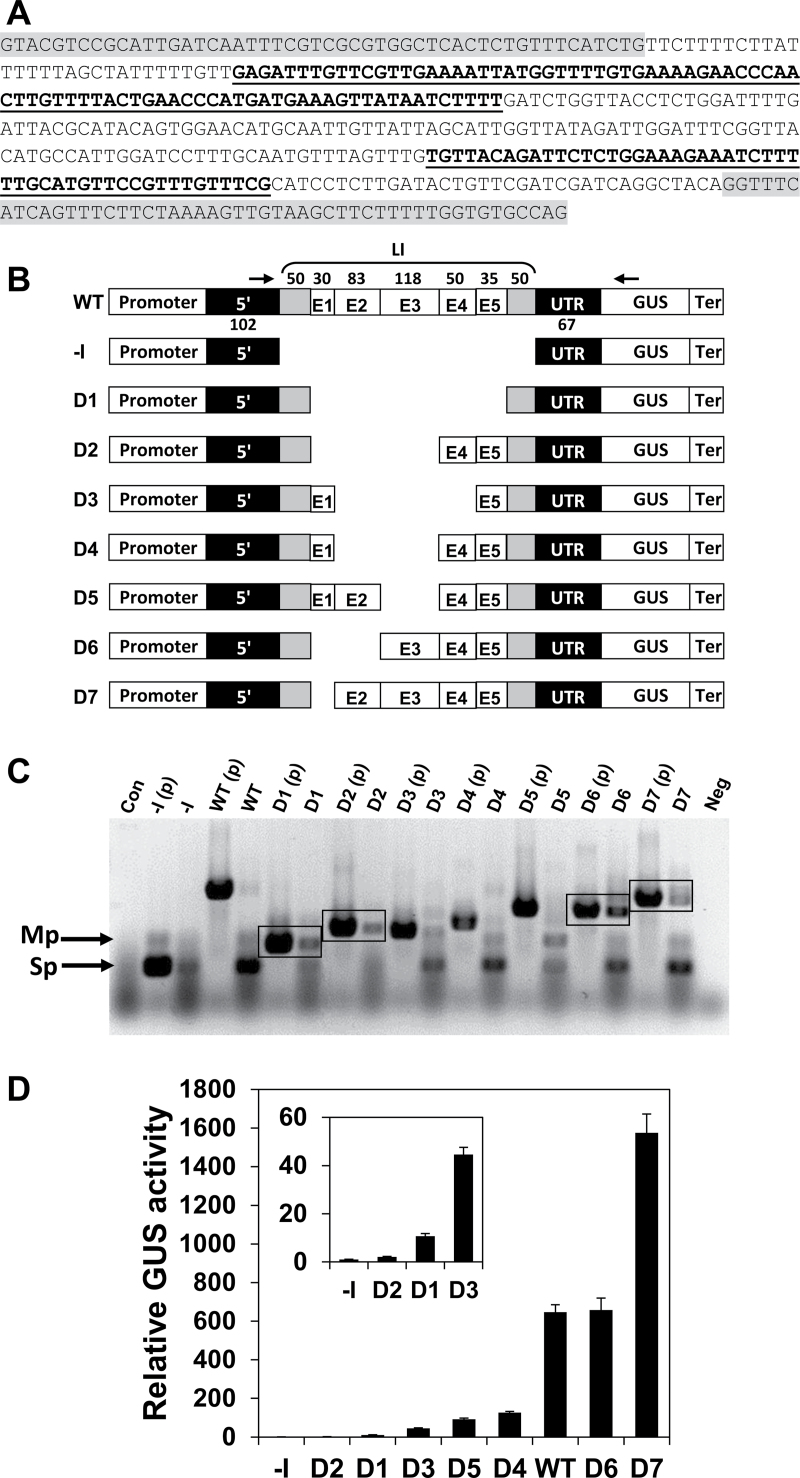
Deletion analysis of the LI. (A) The LI sequence. The two terminal 50 nucleotide regions are highlighted in grey. Elements E1, E3, and E5 are in regular letters. Elements E2 and E4 are indicated by bold, underlined letters. (B) Schematic representation of the DEL series constructs. The promoter, 5′ UTR, and terminator were derived from the *AtMHX* gene. The length (in nucleotides) of each LI element is indicated above the WT construct. The lengths of the two parts of the 5′ UTR located upstream and downstream of the LI are indicated below the WT construct. The arrows indicate the position of the primers used for the analysis shown in (C) (see also the Materials and methods). (C) RT–PCR analysis of plants expressing the DEL series constructs. The PCR products of the intron-containing plasmids [indicated by (p) following the construct name] show the expected sizes of unspliced transcripts. Con, a control PCR using cDNA derived from wild-type, non-transformed *Arabidopsis* plants as a template; Neg, a negative control PCR including all components except the template; Sp, the main spliced product; Mp, minor product of the spliced transcript. The boxes indicate constructs having a relatively higher proportion of unspliced to spliced transcripts. (D) Relative GUS activity of plants expressing the DEL series constructs. Each column shows the mean and standard error of 10 samples, and each sample included ~70 two-week-old plants. GUS activity of the –I construct of the DEL series was assigned the value of 1. The inset graph shows the relative GUS activity compared with the –I construct on a smaller scale in plants expressing the constructs that mediated low GUS activity.

The significance of various internal regions of the LI of *AtMHX* for its ability to enhance expression was first investigated by deletion analysis. The internal LI sequence (excluding the two terminal 50bp regions) was divided into five regions (Fig. 1A). The two terminal 50bp regions were retained in all constructs in order to keep the splice sites and essential neighbouring sequences intact. The first internal region (E1) was a U-rich 30bp element, having a U content of 73%. The rest of the internal sequence was roughly divided into four regions: E2, E3, E4, and E5 (Fig. 1A), considering the length and IMEter scores of these regions (Supplementary Table S1 at *JXB* online). A series of constructs named D1–D7, in which single or multiple internal LI regions were deleted, was created from a basic construct called WT (Fig. 1B). The WT construct included the native promoter, 5′ UTR, LI, and terminator of the *AtMHX* gene, and the coding sequence of the GUS reporter protein. One construct (–I, for minus intron) did not include the LI. This series of constructs is referred to here as the DEL series.

The constructs were transformed into *Arabidopsis.* About 45 independent T_1_ plants were collected for each construct used in this study to compensate the position effect and ensure reliable results. Since splicing is important for efficient IME, RT–PCR analysis was carried out to determine if the deleted introns were correctly spliced. The expected size of the PCR product of a spliced transcript (indicated by the letters Sp in Fig. 1C) is similar to that of the major product of the plasmid carrying the –I construct. Except for D2, the cDNA of all constructs gave rise to PCR products whose size was similar to that of the –I plasmid, indicating that they were at least partially spliced. Note that besides the expected product, the –I plasmid also gave rise to a minor product (Mp). A band with a similar size was also derived from the cDNA of most spliced constructs, but not from their plasmids that contained the unspliced sequences. Since this band also appeared as a by-product of the –I plasmid, it was apparently a non-specific product of the spliced transcript, and not an unspliced isoform. The cDNA of some constructs also showed a PCR product with a size similar to that of their intron-containing plasmids, indicating that they were not spliced at 100% efficiency. Experiments in which different plasmids were mixed indicated that under the PCR conditions used, the spliced and unspliced transcripts co-amplified with equal efficiency. Consequently, the ratio of correctly spliced to total *GUS* transcript of a given construct reflected splicing efficiency. Note that the comparison between different constructs is qualitative and not quantitative (i.e. band intensities do not reflect the differences in transcript levels between different constructs). Unspliced products were particularly noticeable in constructs that lacked the intronic E1 region (constructs D1, D2, D6, and D7; see boxed PCR products in Fig. 1C), but were absent or relatively weak in constructs that included E1 (WT, D3, D4, and D5). For example, D7, in which only E1 was deleted, had a higher ratio of non-spliced to spliced transcript compared with the WT construct. These data indicate that the E1 element increases the efficiency with which the LI is spliced.

GUS activity of plants expressing the different constructs, relative to that of plants expressing the –I construct, is shown in Fig. 1D (the constructs are arranged in the order of their increasing levels of GUS activity). Each column presents the average of 10 samples, and each of the 10 samples included ~70 two-week-old plants (therefore, each column represents the average activity of 700 plants that were germinated from a mixture including an equal number of seeds from each of the 45 T_1_ plants of the indicated construct). Interestingly, plants expressing constructs WT, D6, and D7, which were the only constructs that included the intronic region E3, showed significantly higher GUS activity levels compared with the other constructs. Deletion of E3 alone from the WT construct resulted in a 7-fold reduction in GUS activity (compare constructs WT and D5). This suggests that E3 makes a major contribution to the enhancement capacity of the LI. As shown in Fig. 1C, E3 was not essential for splicing, since constructs D3, D4, and D5, which lacked E3 but included E1, showed lower proportions of unspliced transcript compared with other constructs. Notably, the GUS activity of plants expressing construct D7 was 2.4-fold higher than that of plants expressing the WT construct. This indicates that although E1 enhances the efficiency with which the LI is spliced, it has a negative impact on the ability of this LI to enhance expression.

### Investigating the role of LI elements by a gain-of-function approach

To explore further the role of the various LI regions, a gain-of-function approach was used. Individual or several LI elements were introduced into the middle of a non-related intron predicted to have a relatively low ability to enhance expression. The intron used was derived from the *Ricinus communis CAT1* gene, and was chosen because of its relatively low IMEter score and also because it was used as a foreign intron (that was introduced into *GUS* to avoid expression in bacterial cells) in the pCAMBIA series of plant vectors. All AUG triplets were eliminated (by keeping the same nucleotides in a different order) from both the *CAT1* intron and the LI of *AtMHX*, in order to eliminate the possibility of nonsense-mediated mRNA decay (NMD) or translational inhibition in the case of incomplete splicing. In brief, upstream AUG (uAUG) codons that are recognized by the ribosome (depending on their sequence context) may lead to transcript degradation by the NMD pathway, and/or translational inhibition (Kozak, 2002; Chang *et al.*, 2007; Saul *et al.*, 2009). To simplify the experimental system, the uAUG codon residing in the 5′ UTR of *AtMHX* outside the LI was also eliminated in this series of constructs. These modifications are detailed in the Materials and methods and in Supplementary Figs S1 and S2 at *JXB* online, and were also introduced into the –I (minus intron) construct of the gain-of-function series of constructs. The modifications almost did not alter the IMEter scores of the introns (Supplementary Table S2 at *JXB *online).

**Fig. 2.**
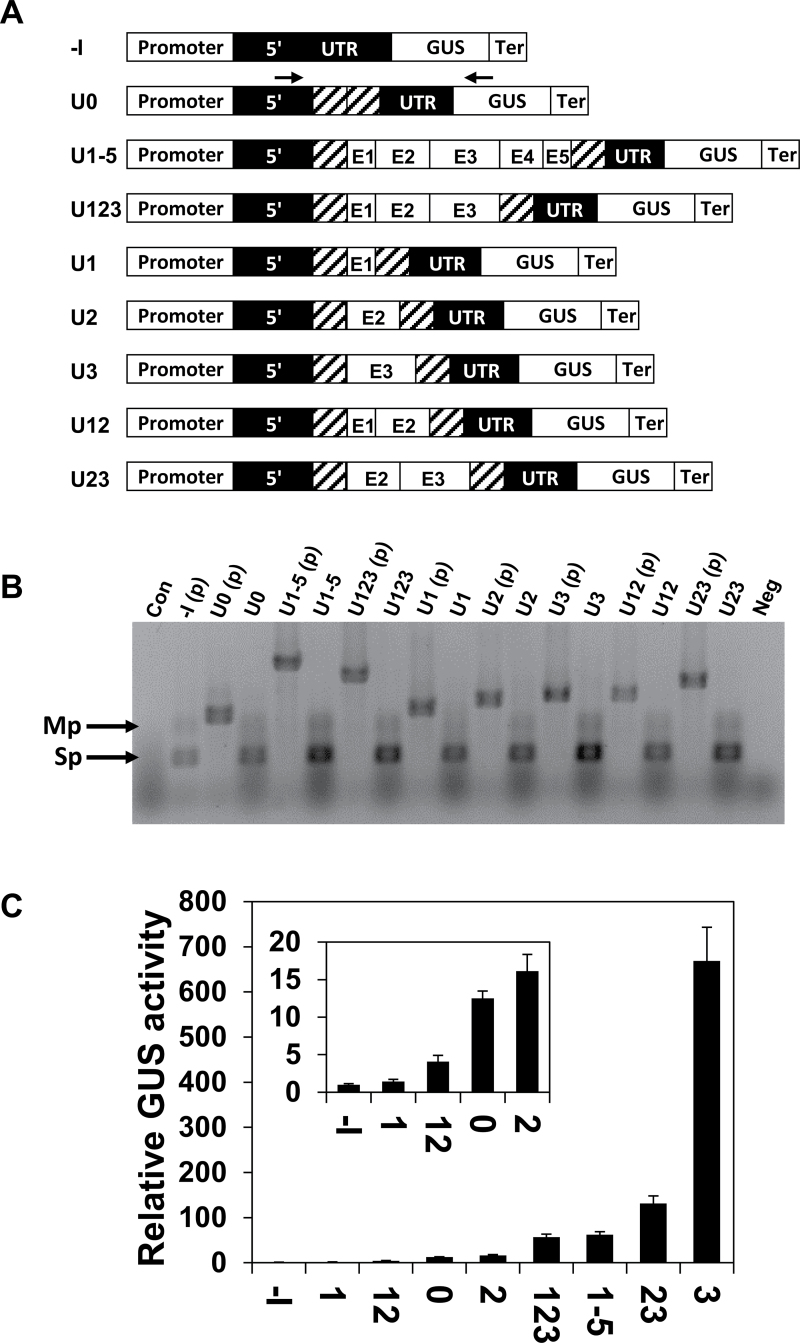
GUS activity and splicing efficiency in plants expressing the UTR series of constructs. (A) Schematic representation of the UTR series constructs. The diagonal lines represent the *CAT1* intron. The arrows on the U0 construct indicate the position of the primers used for the analysis shown in (B). (B) RT–PCR analysis of plants expressing the UTR series constructs. See the legend of Fig. 1C for more details. (C) Relative GUS activity of plants expressing the UTR series constructs. Each column shows the mean and standard error of 10 samples. Each of the 10 samples included ~70 two-week-old plants. GUS activity of the –I construct of the UTR series was assigned the value of 1. The inset graph shows the relative GUS activity compared with the –I construct on a smaller scale.

In the so-called UTR series of gain-of-function constructs, the *CAT1* intron alone (construct U0) or including various LI elements, was used to replace the LI in the 5′ UTR of *AtMHX* (Fig. 2A). For clarity, each construct was prefixed with the letter U (indicating that it belonged to the UTR series) followed by the numbers of the LI elements it included. Regions E1, E2, E3, or E4+E5 (that were treated here as one region) were introduced in various combinations into the middle of the 221bp *CAT1* intron, without deleting any part of this intron. Following transformation into *Arabidopsis*, RT–PCR analysis was carried out. All transformed plants showed PCR products similar to both the major (Sp) and minor (Mp) products of the plasmid carrying the construct that lacked any intron [–I(p)], and did not show a product whose size was similar to that of their unspliced plasmid. This indicated that all introns were spliced with high efficiency, apparently because the *CAT1* intron provided all the necessary signals and probably due to the *a priori* elimination of potential cryptic splice sites (see the Materials and methods, and Supplementary Figs S1 and S2 at *JXB* online). GUS activity of the transformed plants, relative to that of the –I plants, is shown in Fig. 2C (note the smaller scale of the inset graph). All four constructs that had relatively higher activity (U3, U23, U1-5, and U123) included the E3 region, while this region was absent from the other constructs. The inclusion of E3 alone inside the *CAT1* intron resulted in the highest enhancement of GUS activity—670- or 53-fold—compared with the –I or U0 constructs, respectively. This indicated that E3 has a special ability to enhance expression, in agreement with the results of the DEL series. For all constructs used in this study in which the expression level was above the limit of histochemical detection, the sites of GUS expression were similar to those previously reported for the WT construct (David-Assael *et al.*, 2006), and only the intensity of GUS staining differed (data not shown).

The results of the UTR series also supported the conclusion obtained from the DEL series regarding the inhibitory impact of the E1 region. E1 lowered GUS activity when added to the *CAT1* intron alone (U1 to U0 ratio of 0.11), or to the *CAT1* intron that included either E2 (U12 to U2 ratio of 0.25) or E2+E3 (U123 to U23 ratio of 0.43). The strong inhibition seen when E1 was inserted into the *CAT1* intron alone indicated that the negative impact of E1 in the UTR series was not restricted to its interaction with other regions of the LI of *AtMHX*. The function of the E2 region was less clear—its inclusion almost did not affect the *CAT1* intron alone (compare U2 and U0), had a positive effect when added to the inhibitory El element (U12 versus U1), and had a strong negative effect when added to the enhancing E3 region (U23 versus U3). It therefore seems that the positive or negative impact of certain intronic regions on IME can be compensated for by the presence of other intronic elements.

### Element E3 has a special ability to enhance translational efficiency

**Fig. 3.**
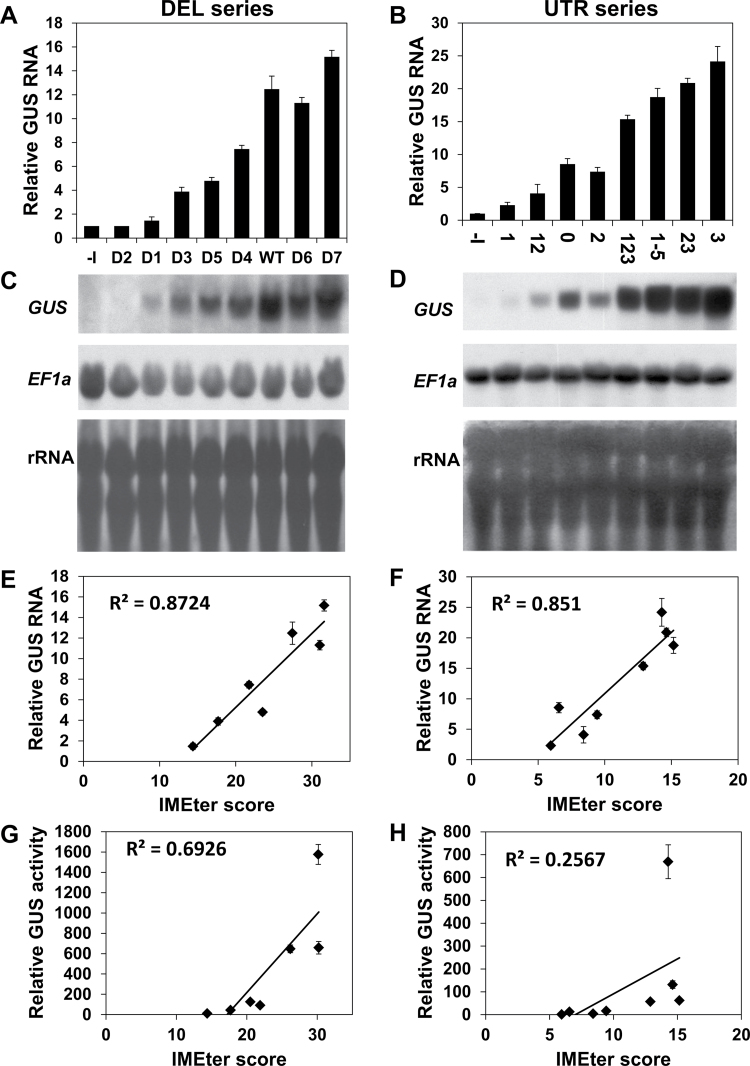
The levels of *GUS* mRNA in plants expressing the DEL and UTR series of constructs. (A and B) Relative levels of *GUS* mRNA in plants expressing the DEL (A) or UTR (B) series constructs. Each column shows the mean and standard error of *GUS* transcript levels in six of the 10 samples whose GUS activity was presented in Figs 1D or 2C, respectively. Each of the six samples included ~70 two-week-old plants (therefore, each column represents the average activity of 420 plants that were germinated from a mixture including an equal number of seeds from each of the 45 T_1_ plants of the indicated construct). *GUS* mRNA content of the –I construct of each series was assigned the value of 1. Quantification of band densities was performed with the ImageJ program (NIH). *GUS* levels in constructs that lacked introns or had a low level of expression were quantified in gels exposed for longer periods (e.g. Supplementary Fig. S4 at *JXB* online). (C and D) Below each graph is one of the six replicates of the northern blot analyses of the DEL (C) or UTR (D) series. Each membrane was first stained with methylene blue to visualize the rRNA in order to confirm equal loading, then probed with the *GUS* coding sequence, and then stripped and probed with the housekeeping *EF1α* gene as another control. (E and F) The graphs show the correlation between *GUS* mRNA levels and the IMEter scores of the DEL (E) or UTR (F) series introns. The IMEter scores were calculated for the whole introns (including the *CAT1* sequence when present) with the second version of the IMEter algorithm (http://korflab.ucdavis.edu/cgi-bin/web-imeter2.pl). The second version of this algorithm was found to be a better predictor than the first version of how well any intron will enhance mRNA accumulation (Parra *et al.*, 2011). Construct D2 was not included in the correlations because it was apparently not spliced. (G and H) The correlation between GUS activity and the IMEter v2.0 scores of the DEL and UTR series introns, respectively.

Introns can enhance gene expression on several levels, including the enhancement of transcript accumulation and the increase in translational efficiency (see the Introduction). In order to understand how the LI elements exert their impact, the levels of *GUS* transcript were determined in plants expressing the DEL and UTR series of constructs (Fig. 3). Each column in Fig. 3A and B shows the average *GUS* transcript levels of six samples, and each of these six samples included RNA derived from a mixture of ~70 T_2_ plants. Figure 3C and D shows one of the six replicates of each series. The constructs were arranged in Fig. 3A–D according to the order of the corresponding constructs in Figs 1D and 2C. In the latter figures, the constructs were presented according to their increasing GUS activity. Thus, the results presented in Fig. 3A and B indicated that, in general, increased GUS activity was correlated with higher steady-state levels of *GUS* mRNA.

**Fig. 4.**
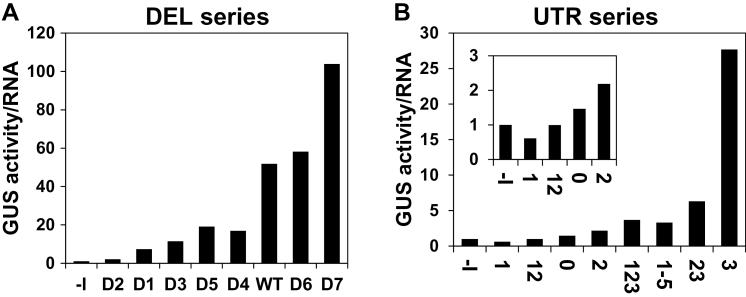
Translational efficiency of the DEL (A) and UTR (B) series of constructs. Each column presents the ratio of average GUS activity to average *GUS* mRNA in the six samples described in Fig. 6A and B. The translational efficiency of the –I construct of each series had the value of 1. The inset graph shows the ratios on a smaller scale.

It was then examined to what extent the levels of *GUS* mRNA or GUS activity correlated with the IMEter scores of the different introns. The levels of *GUS* mRNA showed high correlation with the IMEter scores (Fig. 3E, F). These results are in agreement with the fact that the IMEter score was presented as a value that correlated with the enhancement of mRNA accumulation (Rose *et al.*, 2008; Parra *et al.*, 2011). However, there was only a moderate (Fig. 3G) or weak (Fig. 3H) correlation between the IMEter scores and the levels of GUS activity of the DEL and UTR series, respectively. To determine if the LI elements also enhanced expression at the translational level, the ratio between GUS activity (making the reasonable assumption that GUS activity is proportional to the amount of GUS protein) and *GUS* mRNA was calculated (Fig. 4). This mode of calculating the translational efficiency was commonly used in other studies (e.g. Mascarenhas *et al.*, 1990; Nott *et al.*, 2003, 2004; Rose, 2004). Figure 4 shows that different LI elements increased to different extents the yield of GUS protein for a given transcript content. Similar ratios between GUS activity and *GUS* mRNA were obtained for the same constructs in other independent experiments (data not shown). These data indicate that the investigated LI elements differ in their ability to enhance translation.

The E3 element made a large contribution to the translational efficiency in both series of constructs. In the DEL series, its omission in D5 resulted in a 2.7-fold reduction in translational efficiency compared with the WT construct (Fig. 4A). In the UTR series, the addition of E3 to the *CAT1* intron alone resulted in a 19-fold increase in translational efficiency (Fig. 4B, compare constructs U3 and U0). This indicates that the E3 element has a considerable ability to enhance translation. The addition of E3 to E2 (U23 versus U2), or to E1+E2 (U123 versus U12), increased the translational efficiency 2.9- or 3.7-fold, respectively. However, these data also indicate that the addition of elements E2 (U23 versus U3) or E1+E2 (U123 versus U3) to E3 lowered the ability of E3 to enhance translation by 6.5- or 5-fold, respectively. At the same time, the addition of elements E4+E5 to construct U123 to create U1-5 did not result in a significant reduction in translational efficiency. It is assumed that E3 made the major contribution to the translational efficiency of construct U123, since elements E1+E2 were unable to increase the translational efficiency mediated by the *CAT1* intron (U12 versus U0). Altogether, these data show that the ability of E3 to enhance translation was abrogated by the presence of elements E2 or E1+E2 at its 5′ end, but not by the presence of elements E4+E5 at its 3′ end.

To determine whether the strong (5- or 6-fold) inhibitory impact of E2 or E1+E2 on E3-mediated translational efficiency is related to a specific interaction between these elements and E3, it is necessary to evaluate the effect of the indicated elements in constructs that lack E3. Figure 4B shows that elements E2, or E1+E2, had only a small (~30%) stimulatory or inhibitory effect, respectively, on the translation efficiency mediated by the *CAT1* intron (for E2, compare U2 with U0, and for E1+E2, compare U12 with U0). It thus seems that the indicated inhibitory impact results from interaction between element E3 and elements E2 or E1+E2 (or between putative proteins or RNAs that bind these elements).

The results presented before showed that element E1 inhibited expression in both the DEL and UTR series. The present data show that E1 decreases the ability of introns that include it to enhance translation. There was a 2-fold increase in translational efficiency in construct D7, in which E1 was removed, compared with the WT construct (Fig. 4A). At the same time, there was only a minor (if any) increase in translational efficiency in construct D6, in which both E1 and E2 were removed, compared with the WT construct (Fig. 4A). However, it should be considered that the splicing efficiency (as reflected by the ratio of spliced to total transcript) of construct D6 was about half that of the WT construct (Fig. 1C). The sizes of the spliced and unspliced transcripts of D6 are 2460bp and 2760bp, respectively. Therefore, these transcripts are expected to appear as a single band in the hybridization. The retention of the unspliced intron in the 5′ UTR of D6 may reduce the translational yield of this construct, since the efficiency of translation initiation depends on the length and secondary structure of the 5′ UTR (Kozak, 2002). Thus, the impact of E1 alone could be better evaluated in the UTR series of constructs, in which splicing efficiency was high. In the UTR series, E1 lowered the translational efficiency ~2-fold when added to either the *CAT1* intron alone (Fig. 4B, U1 versus U0) or the *CAT1* intron that included E2 (U12 versus U2) or E2+E3 (U123 versus U23).

### Downstream displacement of the introns into the coding sequence had a stronger inhibitory impact on translational efficiency than on mRNA content

**Fig. 5.**
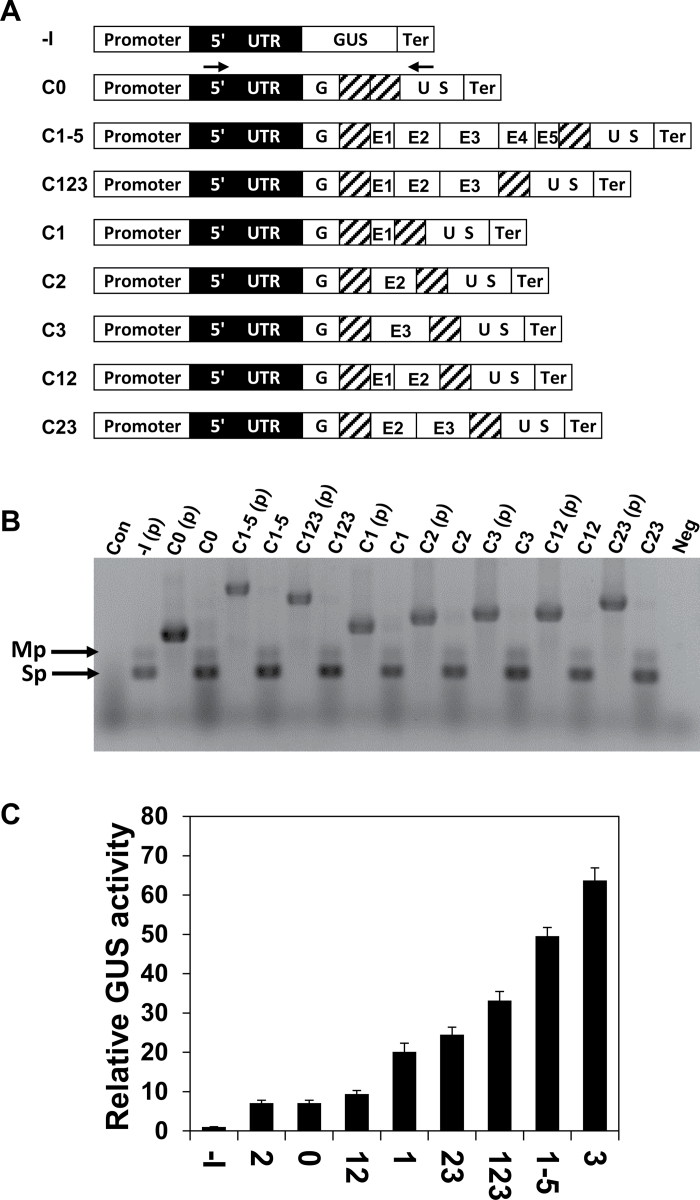
GUS activity and splicing efficiency in plants expressing the CDS series constructs. (A) Schematic representation of the CDS series constructs. The diagonal lines represent the *CAT1* intron. The arrows on the C0 construct indicate the position of the primers used for the analysis shown in (B). (B) RT–PCR analysis of plants expressing the CDS series constructs. See the legend of Fig. 1C for more details. (C) Relative GUS activity of plants expressing the CDS series constructs. Each column shows the mean and standard error of 10 samples. Each of the 10 samples included ~70 two-week-old plants. GUS activity of the –I construct of this series was assigned the value of 1.

The significance of intron position for the function of the LI elements was investigated by moving the same introns used in the UTR series into the coding sequence. The introns were inserted only 12bp downstream to the *GUS* initiation codon; that is, 82bp downstream of their position in the UTR series (Supplementary Figs S1, S3 at *JXB* online). The resulting series of constructs, called CDS, is illustrated in Fig. 5A. The *CAT1* intron was inserted into the *GUS* coding sequence at essentially the same position used in the pCAMBIA vectors. The CDS and UTR series constructs were identical with respect to all sequence elements, and differed only in the position of their introns. Following transformation into *Arabidopsis*, RT–PCR showed that all constructs were spliced at high efficiency (Fig. 5B).

Analysis of GUS activity of the transformed plants indicated that the insertion of E3 alone into the *CAT1* intron resulted in the highest enhancement of expression—64- and 9-fold—compared with the constructs that lacked the intron (–I) or included the *CAT1* intron alone (C0), respectively (Fig. 5C). The inclusion of additional LI elements lowered the ability of E3 to enhance expression. These findings are in agreement with the results of the previous series. Similar to the UTR series, inclusion of E2 alone in the *CAT1* intron did not have a considerable impact on expression (Fig. 5C, compare C2 and C0), but E2 addition to E3 considerably decreased expression (C23 versus C3).

However, in contrast to the findings in both the DEL and UTR series, in which E1 inhibited expression, in the CDS series this element enhanced expression when positioned in the *CAT1* intron alone (C1 versus C0), and had a minor positive impact (or at least did not have a negative impact) when included together with E2 (C12 versus C2) or E2+E3 (C123 versus C23). The fact that a positive, or at least lack of negative, effect of E1 was observed here for three construct pairs that differed only in this element strengthened the conclusion that the impact of E1 differed between the CDS series and the two 5′ UTR-localized series of constructs.

**Fig. 6.**
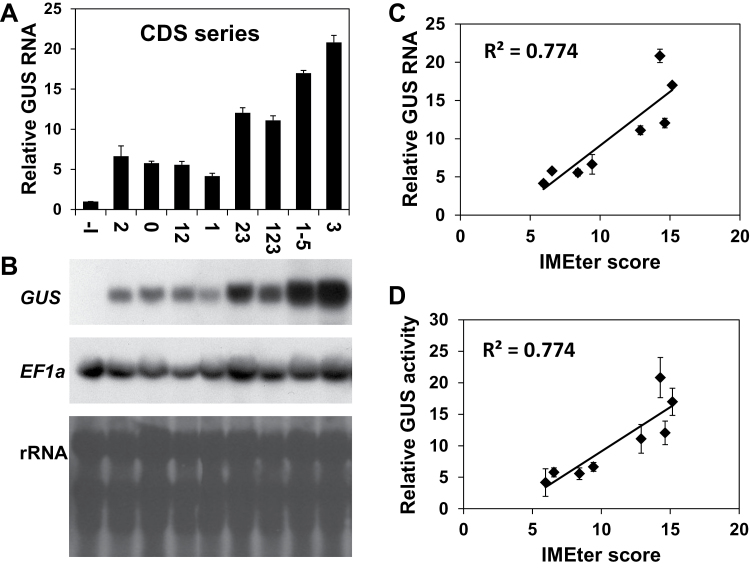
The levels of *GUS* mRNA in plants expressing the CDS series constructs. (A) Relative levels of *GUS* mRNA in plants expressing the CDS series constructs. Each column shows the mean and standard error of *GUS* transcript levels in six of the 10 samples whose GUS activity was presented in Fig. 5C. Each of the six samples included ~70 two-week-old plants. *GUS* mRNA content of the –I construct of this series was assigned the value of 1. (B) One of the six replicates of the northern blot analysis. The membrane was first stained with methylene blue to visualize the rRNA in order to confirm equal loading, then probed with the *GUS* coding sequence, and then stripped and probed with the housekeeping *EF1α* gene as another control. (C) The correlation between *GUS* mRNA levels and the IMEter v2.0 scores of the introns. (D) The correlation between GUS activity and the IMEter v2.0 scores of the introns.

**Fig. 7.**
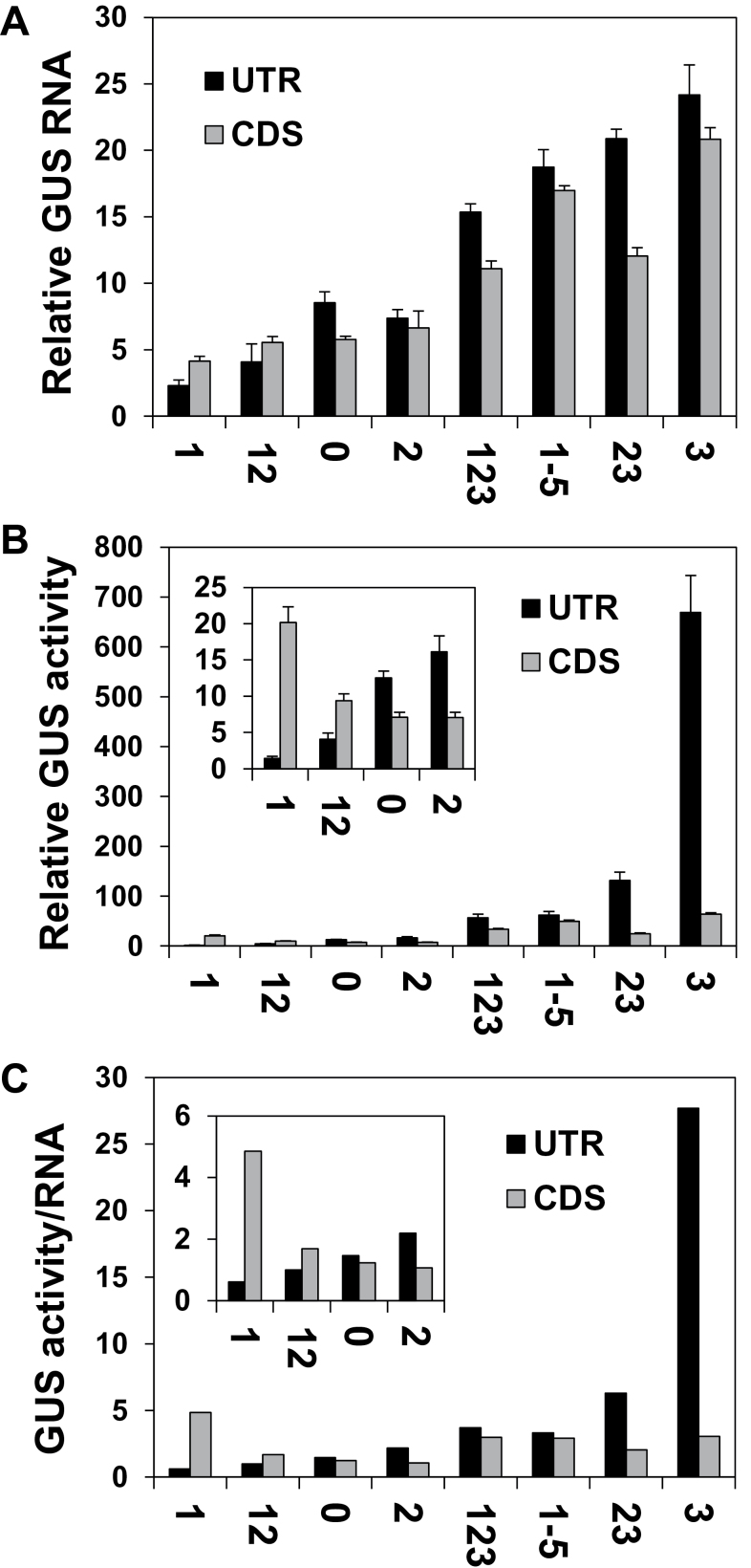
Comparison between the results of the UTR and CDS series. The graphs show the relative levels of *GUS* mRNA (A), GUS activity (B), or translational efficiency (C) of pairs of constructs having the same introns but belonging to the UTR (black columns) or CDS (grey columns) series. The translational efficiency of the –I construct (that was similar in the two series) had the value of 1. The values were calculated as detailed in the previous figures. The inset graphs have smaller scales.

The levels of *GUS* mRNA in plants expressing CDS series constructs is shown in Fig. 6A and B. The plants are presented in the same order as in Fig. 5C (i.e. according to their increasing GUS activity). The levels of both *GUS* mRNA and GUS activity showed relatively good correlation with the IMEter scores of the different introns (Fig. 6C, D). To better evaluate the consequences of the change in intron position, the results of the UTR and CDS series were compared. Figure 7A and B shows the levels of *GUS* mRNA and GUS activity of pairs of constructs having the same intron in different positions—either the 5′ UTR (black columns) or the coding sequence (grey columns) (the constructs were presented according to the increasing GUS activity of the UTR series). All data were calculated relative to the same construct (–I) and, consequently, the difference in column lengths between each pair of constructs is proportional to the difference in the absolute levels of *GUS* mRNA or activity between the two constructs.

Figure 7A indicates that the absolute levels of *GUS* mRNA were, in general, comparable in constructs having the same intron at the two different locations. Still, for most construct pairs, mRNA levels were 10–40% lower in the CDS series. It was shown that the ability to enhance mRNA accumulation is proportional to the intron’s proximity to the 5′ end of the transcript (Rose, 2004, and references therein). The extent of the reduction observed here in mRNA content is in line with previous reports in which 300bp downstream displacements of the first introns of *AtTRP1* or *AtUBQ10* decreased mRNA levels by 30% only (Rose, 2004). It is therefore reasonable that the downstream displacement of only 82bp carried out here resulted in a relatively small decrease in transcript levels. However, this 82bp displacement resulted in a relatively large reduction in the ability of some of the investigated introns to elevate GUS activity (Fig. 7B), indicating that there was a relatively large reduction in the ability of these introns to enhance translation (Fig. 7C). Specifically, the introns of constructs C3, C23, and C2 had a 9-, 3-, or 2-fold lower ability to enhance translation compared with the same introns in constructs U3, U23, and U2, respectively (Fig. 7C). The same displacement of the *CAT1* intron alone almost did not affect its ability to enhance translation, which was rather low in both the UTR and CDS series (constructs U0 and C0 mediated 1.47- and 1.23-fold translational enhancements, respectively). This indicates that the reduction in the ability to enhance translation due to intron displacement in constructs C3, C23, and C2 primarily resulted from the shift in E3 and E2 location.

Element E1 alone had a much higher ability to enhance translation in the CDS than in the UTR series (Fig. 7C, C1 versus U1). This may explain the observations that (i) translational enhancement mediated by E1+E2 was higher in the CDS than in the UTR series (Fig. 7C, C12 versus U12); and (ii) although E2+E3-mediated translational enhancement was lower in the CDS than in the UTR series (C23 versus U23), translation enhancement was similar in the two series for constructs C1-5–U1-5 and C123–U123, which included E1 in addition to E2+E3.

Altogether, these data show that a 82bp downstream shift of introns that contained E3 from the 5′ UTR into the coding sequence resulted in a minor decrease in mRNA accumulation, but in a major drop in the ability of E3 to enhance translation.

## Discussion

Deletion analysis studies carried out thus far were unable to identify specific intronic regions essential for IME (reviewed by Rose, 2008). The short IMEter sequences that increased mRNA content were shown to be redundant and dispersed throughout enhancing introns. Consequently, the ability to boost expression of entire introns was found to be roughly the sum of the stimulation mediated by each of their parts (Rose *et al.*, 2008). As far as is known, this work presents the first identification of an internal intronic region (the E3 element) that has a special ability to enhance translational efficiency, without being essential for splicing. The analysis also showed that the impact of E3 on translational efficiency was repressed by other intronic elements. Such interference between neighbouring intronic elements had not been reported thus far. In addition, this work showed that the ability of E3 to enhance translation was strongly dependent on its localization in a 5′ UTR intron. The 82bp downstream displacement of the introns of constructs U3 or U23 from the 5′ UTR into the coding sequence diminished their ability to enhance translation. This suggests that 5′ UTR introns provide a preferable platform over downstream introns for elements engaged in enhancing translational efficiency. These findings provide experimental support for some of the bioinformatics-based ideas that were raised to explain the extra length of 5′ UTR introns (see the Introduction). As mentioned, it is currently unknown why 5′ UTR introns are considerably longer than first introns of genes that lack 5′ UTR introns (Bradnam and Korf, 2008). Calculations indicated that the IMEter sequences could not account for all of this extra length (Bradnam and Korf, 2008). It is possible that some 5′ UTR introns include transcription-enhancing elements, as demonstrated for the rice *Ostub16* gene (Morello *et al.*, 2002). However, as detailed in the Introduction, many introns, including the LI of AtMHX, do not enhance expression by acting as transcriptional enhancers. The current work suggests that some of the extra length of 5′ UTR introns results from the presence of translation-enhancing elements, and from the significance of localization in the 5′ UTR for the ability of these elements to enhance translation.

**Fig. 8.**
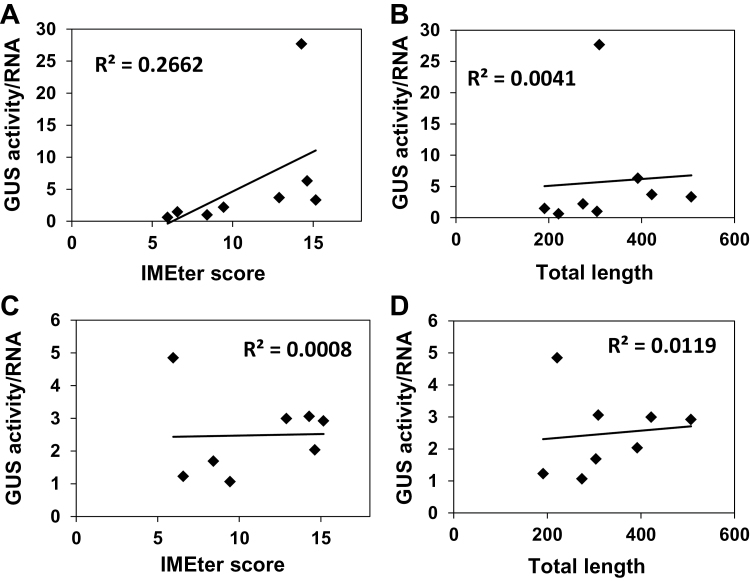
Translational efficiency did not correlate with lengths of the introns or their IMEter scores. Translational efficiency (the ratio of average GUS activity to average *GUS* mRNA in the same samples) of the UTR (A, B) or CDS (C, D) series introns is plotted against the IMEter scores (A, C) or the total lengths (B, D) of the introns.

The abundance of IMEter sequences in different introns (as reflected by the IMEter scores of the introns) was shown to correlate with the extent of increase in mRNA accumulation in *Arabidopsis* (Rose *et al.*, 2008; Parra *et al.*, 2011). For all three series of constructs studied here, the levels of *GUS* mRNA showed high correlation with the IMEter scores of the introns (Figs 3E, F, 6C). This indicated that the impact of the LI elements on mRNA accumulation was related to their content of the IMEter sequences. The enhancement of translational efficiency by the different introns studied did not correlate with the IMEter scores or the total lengths of the introns (Fig. 8). This indicates that the IMEter signals do not affect the efficiency of translation. The impact of the 82bp downstream shift in the location of introns on mRNA accumulation was much smaller than the impact on translational efficiency. This indicates that positioning in the 5′ UTR is not crucial for an intron’s ability to increase the mRNA content, as opposed to an intron’s ability to enhance translation.

Based on bioinformatics, it was also hypothesized that some of the extra length of 5′ UTR introns might be necessary for separating different functional intronic elements, in order to prevent interference between proteins or RNAs that bind them (Bradnam and Korf, 2008). The current work provides experimental support for this hypothesis by showing that the ability of E3 to enhance translation was repressed by neighbouring intronic elements, particularly E1. The U-rich (U content of 73%) E1 element contributed to efficient splicing of the DEL series introns. Uridine (U) residues are important for splicing (reviewed by Lorkovic *et al.*, 2000), and plant U-rich intronic elements were shown to bind the UBP1 protein that enhances splicing efficiency (Lambermon *et al.*, 2000). One putative explanation for the apparent interference between E3 and E1 is that a factor bound by E1 in order to accomplish its role in splicing (such as UBP1) disrupts E3 interaction with factors that mediate the increase in translational efficiency. The possibility that this interference results from the formation of some secondary structures should also be considered. However, the AU content of E3 (63%) is identical to that of the whole LI, and the Mfold server (http://mfold.rna.albany.edu/?q=mfold/RNA-Folding-Form) (Zuker, 2003) did not predict that any of the introns used in this study has distinctive properties in terms of its internal stability or secondary structure (data not shown).

Intriguingly, while E1 lowered translational efficiency in the two 5′ UTR-localized series (i.e. DEL and UTR), it enhanced translation in the CDS series, in which the introns were localized in the coding sequence. The extent of this enhancement (a 4-fold increase in the translational efficiency mediated by the *CAT1* intron alone) was, however, much lower compared with the 19-fold increase mediated by E3 in the UTR series. It is possible that in the CDS series introns, in which the ability of E3 or other intronic elements to enhance translation was diminished (due to the change in their position), the inhibitory impact of E1 on the function of these elements became less significant, and a positive impact of E1 on translation became unmasked. A positive impact of U-rich intronic motifs on IME, without an impact on splicing, had been reported (Clancy and Hannah, 2002).

The specific mechanism by which E3 increases translational efficiency is not clear yet. Although IME was identified in many eukaryotes, very little is currently known about proteins that mediate it. No protein responsible for the IMEter element-mediated increase in mRNA content was identified thus far. Element search in E3 using the RegRNA site (http://regrna.mbc.nctu.edu.tw/) identified only short (3–6 nucleotides) motifs previously shown to affect splicing efficiency or alternative splicing in mammalian cells (data not shown). Since the identified short motifs were implicated in splicing, they are not related to the impact of E3 (which does not have an apparent essential role in splicing) on translational efficiency. It was shown that the enhancing effect of introns on translation is mediated by the EJC (Wiegand *et al.*, 2003; Nott *et al.*, 2004; reviewed by Le Hir *et al.*, 2003; Moore and Proudfoot, 2009). However, only two EJC-associated proteins that enhance translation have been identified thus far—PYM (Diem *et al.*, 2007) and S6K1 (Max *et al.*, 2008) (see the Introduction). The four core EJC proteins are associated with other proteins, which may differ under different conditions (reviewed by Bono and Gehring, 2011). The majority of EJC components are recruited to the spliceosome and interact with the pre-mRNA prior to exon ligation (Jurica *et al.*, 2002; Makarov *et al.*, 2002; Reichert *et al.*, 2002; Merz *et al.*, 2007; Zhang and Krainer, 2007; Gehring *et al.*, 2009a). It was shown that introns themselves play a role in this recruitment—some EJC components are first recruited by intron-associated proteins, and are subsequently translocated to the EJC (Hirose *et al.*, 2006; Ideue *et al.*, 2007). We hypothesize that element E3 binds, either directly or indirectly, some protein that subsequently becomes part of the messenger ribonucleoprotein particle (mRNP; possibly as part of the EJC) that reaches the cytosol, where it functions to enhance translational efficiency. To understand E3 function fully, it will be necessary to determine the core sequence of this element that is required for translation enhancement, to look for proteins that bind this sequence, and to determine if and how E3 affects mRNP composition.

The LI of *AtMHX* was previously shown to enhance the GUS activity mediated by the strong constitutive *Cauliflower mosaic virus* (CaMV) 35S promoter by ~3-fold (Akua *et al.*, 2010). This extent was, however, much lower than the enhancement mediated by this LI when GUS was expressed under the control of the *AtMHX* promoter (Akua *et al.*, 2010). The data presented by Akua *et al.* (2010) showing that GUS mRNA levels were also increased by ~3-fold in plants expressing the 35S construct implicated that the LI of AtMHX did not enhance translation when GUS was expressed under the control of the 35S promoter. One possible explanation is that the enhancement of translation depends on specific factors that are only present in the sites of *AtMHX* expression. However, it should be noted that the 35S promoter is much stronger than the *AtMHX* promoter (Akua *et al.*, 2010). It is therefore possible that when the cellular machinery is occupied with efficiently expressed genes, further enhancement in the efficiency of expression can only be achieved to a limited extent. Further study will be necessary to determine if the ability of introns to enhance translation correlates with promoter strength.

It was previously reported that while *AtMHX* transcript could be easily visualized, expression of the corresponding protein is apparently low (David-Assael *et al.*, 2005). These observations were attributed to the translational repression mediated by the uAUG of *AtMHX* (David-Assael *et al.*, 2005). The current work provides another possible reason for these observations, by suggesting that the efficiency of AtMHX translation is dependent on its 5′ UTR intron. As mentioned, the enhancement of translational efficiency by introns depends on the EJC (Wiegand *et al.*, 2003; Nott *et al.*, 2004), which is removed from the mRNA during the pioneer round of translation. Although the efficiency of the pioneer round of translation influences the efficiency of subsequent rounds (Sato *et al.*, 2008), it was suggested that the enhancement of translational efficiency by introns increases the likelihood of newly exported mRNAs being preferentially translated over older transcripts (Gehring *et al.*, 2009b; Moore and Proudfoot, 2009). This mechanism was suggested to be particularly important for signal transduction and regulatory pathways (Gehring *et al.*, 2009b; Moore and Proudfoot, 2009). Interestingly, genes with regulatory roles are particularly enriched in 5′ UTR introns (Cenik *et al.*, 2010). It will be of interest to determine if long 5′ UTR introns of other eukaryotic genes gained during their evolution elements that enhance translation.

## Supplementary data

Supplementary data are available at *JXB* online.

Figure S1. The sequences of the 5′ UTR of *AtMHX* and the *CAT1* intron.

Figure S2. The modifications introduced into the LI elements in the UTR and CDS series compared with the DEL series.

Figure S3. The coding sequence of *GUS*.

Figure S4. The northern blot shown in Fig. 3D of the manuscript exposed for a longer period.

Table S1. IMEter scores of individual LI elements.

Table S2. IMEter scores of the modified LI elements inside *CAT1* compared with putative similar constructs containing the comparable non-modified LI elements.

Supplementary Data

## Abbreviations:

EJC: exon junction complex
IME: intron-mediated enhancement
LI: leader intron
NMD: nonsense-mediated mRNA decay
RT–PCR: reverse transcription–polymerase chain reaction
uAUG: upstream AUG
5′ UTR: 5′ untranslated region.
