# Supplementary material for: The Arabidopsis thaliana MHX gene includes an intronic element that boosts translation when localized in a 5′ UTR intron
Source: J Exp Bot. 2013 Sep 4;64(14):4255–70. doi: 10.1093/jxb/ert235 (PMC3808313; doi:10.1093/jxb/ert235)
Supplement: Supplementary Data [file supp_64_14_4255__index.html]

The Arabidopsis thaliana MHX gene includes an intronic element that boosts translation when localized in a 5′ UTR intron — Supplementary Data 

# The *Arabidopsis thaliana MHX* gene includes an intronic element that boosts translation when localized in a 5′ UTR intron

## Supplementary Data

Data files

**Files in this Data Supplement:**

- Supplementary Data - Supplementary Data
