# Supplementary material for: The Arabidopsis thaliana MHX gene includes an intronic element that boosts translation when localized in a 5′ UTR intron
Source: J Exp Bot. 2013 Sep 4;64(14):4255–70. doi: 10.1093/jxb/ert235 (PMC3808313; doi:10.1093/jxb/ert235)
Supplement: Supplementary Data [file supp_ert235_ert235_Supplementary_data.pdf]

Supplementary data

The *Arabidopsis MHX* gene includes an intronic element that boosts translation when localized in a 5’ UTR intron

Tsofit Akua and Orit Shaul

Table S1

Element	IMEter v1.0
**E1**	0.831
**E2**	1.665
**E3**	-0.793
**E4**	3.549
**E5**	6.101

Table S1. IMEter scores of individual LI elements.

The scores were calculated (without deleting any nucleotides from the 5' or 3' ends of each element) with IMEter v1.0 (http://korflab.ucdavis.edu/cgi-bin/web-imeter.pl) (only IMEter v1.0 was available when this study was initiated). We divided the internal LI sequence into regions having, as much as possible (considering also the length of each region), roughly comparable IMEter scores.

Table S2

Constructs of the UTR or CDS series	IMEter v2.0 scores of *CAT1* introns containing the modified LI elements	IMEter v2.0 scores of putative *CAT1* introns containing the original LI elements	% Difference in IMEter v2.0 scores of *CAT1* introns containing the modified sequence
**U1 or C1**	5.95	5.95	0
**U0 or C0**	6.56	6.56	0
**U12 or C12**	8.41	9.02	-6.8
**U2 or C2**	9.43	10.13	-6.98
**U123 or C123**	12.89	13.49	-4.48
**U3 or C3**	14.28	14.28	0
**U23 or C23**	14.63	15.33	-4.6
**U1-5 or C1-5**	15.16	16.09	-5.8

Table S2. IMEter scores of the modified LI elements inside the *CAT1* compared to putative similar constructs containing the comparable non-modified LI elements.

The constructs are presented in the order of their increasing IMEter scores. The modifications in the LI sequence resulted in only 4-7% reduction in the IMEter scores of most constructs compared to putative similar constructs containing the comparable non-modified LI elements. The scores of the introns of constructs U1-C1 (in which E1 was not modified) and U3-C3 were identical to that of a putative *CAT1* intron containing the original LI sequence. For constructs U0-C0, which did not include any LI elements, the value reflects the score of the *CAT1* intron alone. The scores were calculated with IMEter v2.0 (http://korflab.ucdavis.edu/cgi-bin/web-imeter2.pl) using the full sequence of each intron, including the *CAT1* sequence. The second version of the IMEter algorithm was found to be a better predictor than the first version of how well any intron will enhance mRNA accumulation (Parra et al., 2011).

Fig. S1

A

```
                                                                 Intron
Origi: 1    AACGCTTGACCGATTCCAATCAGCTCCTCTCGATTTCCGTTTGTCGGAAAATCTCTCCCG  60
            ||||||||||||||||||||||||||||||||||||||||||||||||||||||||||||
Modif: 1    AACGCTTGACCGATTCCAATCAGCTCCTCTCGATTTCCGTTTGTCGGAAAATCTCTCCCG  60

Origi: 61   TGATCGGCGTATTGTGAATGCCGCTCACCGAGATATTCTCCGATTCTTTTCCCCAGTGAG  120
            ||||||||||||||||| |||||||||||||||||||||||| |||||||||||||||||
Modif: 61   TGATCGGCGTATTGTGAAGGCCGCTCACCGAGATATTCTCCGGTTCTTTTCCCCAGTGAG  120

Origi: 121  GACAAGTGTTCAGTTGACTTATTAGGAGGTGGGGTTTGAATAAGTTACC  169
            ||||||||||||||||||||||||||||| |||||||||||||||||||
Modif: 121  GACAAGTGTTCAGTTGACTTATTAGGAGGAGGGGTTTGAATAAGTTACC  169
```

B

```
Origi: 1   gtaaatttctagtttttctccttcattttcttggttaggaccctttctctttttatttt 60
           ||||||||||||||||||||||||||||||||||||||||||||||||||||||||||||
Modif: 1   gtaaatttctagtttttctccttcattttcttggttaggaccctttctctttttatttt 60

                 NheI                                HpaI  AflII
Origi: 61  tttgagctttgatctttctttaaactgatctattttttaattgat-tggttatggtgtaa 119
           ||||||||   |||||||||||||||||||||||||||||||| |    ||| | | |||
Modif: 61  tttgagctagcatctttctttaaactgatctattttttaattgttaaccttaagctctaa 120

                                                   LI elements

Origi: 120 atattacatagctttaactgataatctgattactttatttcgtgtgtctatgatgatgat 179
           |||||||||||||||||||||||||||||||||||||||||||||||||       ||  
Modif: 121 atattacatagctttaactgataatctgattactttatttcgtgtgtctgattgattgga 180

Origi: 180 gatagttacag 190
            ||| ||||||
Modif: 181 aatatttacag 191
```

Fig. S1. The sequences of the 5’ UTR of *AtMHX* and the *CAT1* intron.

(**A**) The modifications introduced into the 5’ UTR of *AtMHX* in the UTR and CDS series compared to the DEL series. Origi: – original sequence, Modif: – modified sequence. The T to G mutation (bold, underlined letter) is for eliminating the upstream AUG codon of the 5’ UTR. The two other modifications (highlighted in gray) are for elimination of potential cryptic splice sites. The position of the LI of *AtMHX* or the *CAT1* intron in the 5’ UTR is indicated by the arrow.

(**B**) The modified *CAT1* intron used throughout this study compared to the original sequence of this intron in *Ricinus communis*. Nucleotides introduced to create restriction enzyme sites are underlined. The modifications indicated by bold letters are for elimination of ATG triplets (by keeping the same nucleotides in a different order). The modifications highlighted in gray are for elimination of potential cryptic splice sites. The position into which the LI elements were subsequently introduced (in the middle of the HpaI site) is indicated by the arrow. Prediction of potential cryptic splice sites was carried out using the NetPlantGene Server (http://www.cbs.dtu.dk/services/NetPGene/).

Fig. S2

```
                                   289 
Origi: 1   gtacgtccgcattgatcaatttcgtcgcgtggctcactctgtttcatctgttcttttctt 60
           ||||||||||||||||||||||||||||||||||||||||||||||||||||||||||||
Modif: 1   gtacgtccgcattgatcaatttcgtcgcgtggctcactctgtttcatctgttcttttctt 60

Origi: 61  atttttagctatttttgttgagatttgttcgttgaaaattatggttttgtgaaaagaac 120
           ||||||||||||||||||||||||||||||||||||||||||  ||||||||||||||||
Modif: 61  atttttagctatttttgttgagatttgttcgttgaaaattagtgttttgtgaaaagaac 120

Origi: 121 ccaacttgttttactgaacccatgatgaaagttataatcttttgatctggttacctctgg 180
           |||||||||||||||||||||  |  ||||||||||||||||||||||||||||||||||
Modif: 121 ccaacttgttttactgaaccctagtagaaagttataatcttttgatctggttacctctgg 180

Origi: 181 atttgattacgcatacagtggaacatgcaattgttattagcattggttatagattggat 240
           ||||||||||||||||||||||||  ||||||||||||||||||||||||||||||||||
Modif: 181 atttgattacgcatacagtggaactagcaattgttattagcattggttatagattggat 240

                                                      289
Origi: 241 ttcggttacatgccattggatcctttgcaatgtttagtttgtgttacagattctctggaa 300
           |||||||||  ||||||||||||||||||  ||||||||||||||||| |||||||||||
Modif: 241 ttcggttactagccattggatcctttgcatagtttagtttgtgttacatattctctggaa 300

Origi: 301 agaaatcttttgcatgttccgtttgtttcgcatcctcttgatactgttcgatcgatcag 360
           |||||||||||||  |||||||||||||||||||||||||||||||||||||||||||||
Modif: 301 agaaatcttttgctagttccgtttgtttcgcatcctcttgatactgttcgatcgatcag 360

                                               396
Origi: 361 gctacaggtttcatcagtttcttctaaaagttgtaagcttctttttggtgtgccag 416
           ||||||||||||||||||||||||||||||||||| ||||||||||||||||||||
Modif: 361 gctacaggtttcatcagtttcttctaaaagttgtacgcttctttttggtgtgccag 416
```

Fig. S2. The modifications introduced into the LI elements in the UTR and CDS series compared to the DEL series.

The different elements were color highlighted as follows: E1 – yellow, E2 – green, E3 – blue, E4 – orange, and E5 – pink. The two terminal 50 nt regions were highlighted in gray. Origi: – original sequence. Modif: – modified sequence. The modifications indicated by bold, underlined letters were for elimination of all ATG triplets (by keeping the same nucleotides in a different order). The G to T substitution at position 289 was for elimination of a potential cryptic splice site. The A to C substitution at position 396 was for elimination of a HindIII site that interfered with cloning into the binary vector. The modifications resulted in less than 7% reduction in the IMEter scores of the LI elements (Supplemental Table 2).

Fig. S3

```
Intron
   ↓
ATGGTAGACCTGAGGAACCGACGACTCGTCCGTCCTGTAGAAACCCCAACCCGTGAAATCAAAAAACTCGACGGCCT
GTGGGCATTCAGTCTGGATCGCGAAAACTGTGGAATTGATCAGCGTTGGTGGGAAAGCGCGTTACAAGAAAGCCGGG
CAATTGCTGTGCCAGGCAGTTTTAACGATCAGTTCGCCGATGCAGATATTCGTAATTATGCGGGCAACGTCTGGTAT
CAGCGCGAAGTCTTTATACCGAAAGGTTGGGCAGGCCAGCGTATCGTGCTGCGTTTCGATGCGGTCACTCATTACGG
CAAAGTGTGGGTCAATAATCAGGAAGTGATGGAGCATCAGGGCGGCTATACGCCATTTGAAGCCGATGTCACGCCGT
ATGTTATTGCCGGGAAAAGTGTACGTATCACCGTTTGTGTGAACAACGAACTGAACTGGCAGACTATCCCGCCGGGA
ATGGTGATTACCGACGAAAACGGCAAGAAAAAGCAGTCTTACTTCCATGATTTCTTTAACTATGCCGGAATCCATCG
CAGCGTAATGCTCTACACCACGCCGAACACCTGGGTGGACGATATCACCGTGGTGACGCATGTCGCGCAAGACTGTA
ACCACGCGTCTGTTGACTGGCAGGTGGTGGCCAATGGTGATGTCAGCGTTGAACTGCGTGATGCGGATCAACAGGTG
GTTGCAACTGGACAAGGCACTAGCGGGACTTTGCAAGTGGTGAATCCGCACCTCTGGCAACCGGGTGAAGGTTATCT
CTATGAACTCGAAGTCACAGCCAAAAGCCAGACAGAGTCTGATATCTACCCGCTTCGCGTCGGCATCCGGTCAGTGG
CAGTGAAGGGCCAACAGTTCCTGATTAACCACAAACCGTTCTACTTTACTGGCTTTGGTCGTCATGAAGATGCGGAC
TTACGTGGCAAAGGATTCGATAACGTGCTGATGGTGCACGACCACGCATTAATGGACTGGATTGGGGCCAACTCCTA
CCGTACCTCGCATTACCCTTACGCTGAAGAGATGCTCGACTGGGCAGATGAACATGGCATCGTGGTGATTGATGAAA
CTGCTGCTGTCGGCTTTCAGCTGTCTTTAGGCATTGGTTTCGAAGCGGGCAACAAGCCGAAAGAACTGTACAGCGAA
GAGGCAGTCAACGGGGAAACTCAGCAAGCGCACTTACAGGCGATTAAAGAGCTGATAGCGCGTGACAAAAACCACCC
AAGCGTGGTGATGTGGAGTATTGCCAACGAACCGGATACCCGTCCGCAAGGTGCACGGGAATATTTCGCGCCACTGG
CGGAAGCAACGCGTAAACTCGACCCGACGCGTCCGATCACCTGCGTCAATGTAATGTTCTGCGACGCTCACACCGAT
ACCATCAGCGATCTCTTTGATGTGCTGTGCCTGAACCGTTATTACGGATGGTATGTCCAAAGCGGCGATTTGGAAAC
GGCAGAGAAGGTACTGGAAAAAGAACTTCTGGCCTGGCAGGAGAAACTGCATCAGCCGATTATCATCACCGAATACG
GCGTGGATACGTTAGCCGGGCTGCACTCAATGTACACCGACATGTGGAGTGAAGAGTATCAGTGTGCATGGCTGGAT
ATGTATCACCGCGTCTTTGATCGCGTCAGCGCCGTCGTCGGTGAACAGGTATGGAATTTCGCCGATTTTGCGACCTC
GCAAGGCATATTGCGCGTTGGCGGTAACAAGAAAGGGATCTTCACTCGCGACCGCAAACCGAAGTCGGCGGCTTTTC
TGCTGCAAAAACGCTGGACTGGCATGAACTTCGGTGAAAAACCGCAGCAGGGAGGCAAACAATGA
```

Fig. S3. The coding sequence of *GUS*.

The position of the introns introduced in the CDS series is indicated by the arrow. The *CAT1* intron derivates introduced here included elimination of a potential cryptic splice site (Fig. S1B). This elimination was shown to result in full and accurate splicing of this intron when introduced into this position of *GUS* (Ma *et al.*, 2011).

.

Fig. S4

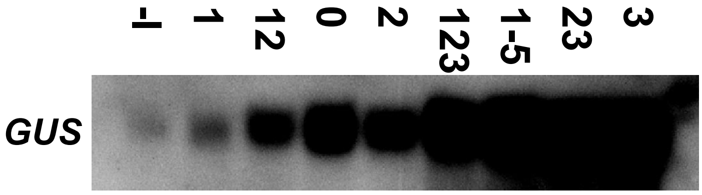

Fig. S4. The Northern blot shown in Fig. 3D of the manuscript exposed for a longer period. The levels of *GUS* mRNA in constructs -I and U1 relative to construct U12 were determined using the ImageJ program (NIH). The values obtained were used to relate *GUS* mRNA levels in the constructs that had low levels of *GUS* mRNA to the values of other constructs, which were quantified from gels exposed for shorter periods.
